# Bruton’s Tyrosine Kinase (BTK) Inhibitors and Autoimmune Diseases: Making Sense of BTK Inhibitor Specificity Profiles and Recent Clinical Trial Successes and Failures

**DOI:** 10.3389/fimmu.2021.662223

**Published:** 2021-11-03

**Authors:** Garth E. Ringheim, Matthew Wampole, Kinsi Oberoi

**Affiliations:** ^1^Clinical Pharmacology and Translational Medicine, Eisai Inc, Woodcliff Lake, NJ, United States; ^2^Science Group, Clarivate, Philadelphia, PA, United States

**Keywords:** Bruton’s tyrosine kinase (BTK), B cell, autoimmune disease, rheumatoid arthritis, systemic lupus erythematosus, multiple sclerosis, pemphigus vulgaris, Sjogren’s syndrome

## Abstract

Clinical development of BTK kinase inhibitors for treating autoimmune diseases has lagged behind development of these drugs for treating cancers, due in part from concerns over the lack of selectivity and associated toxicity profiles of first generation drug candidates when used in the long term treatment of immune mediated diseases. Second generation BTK inhibitors have made great strides in limiting off-target activities for distantly related kinases, though they have had variable success at limiting cross-reactivity within the more closely related TEC family of kinases. We investigated the BTK specificity and toxicity profiles, drug properties, disease associated signaling pathways, clinical indications, and trial successes and failures for the 13 BTK inhibitor drug candidates tested in phase 2 or higher clinical trials representing 7 autoimmune and 2 inflammatory immune-mediated diseases. We focused on rheumatoid arthritis (RA), multiple sclerosis (MS), and systemic lupus erythematosus (SLE) where the majority of BTK nonclinical and clinical studies have been reported, with additional information for pemphigus vulgaris (PV), Sjogren’s disease (SJ), chronic spontaneous urticaria (CSU), graft *versus* host disease (GVHD), and asthma included where available. While improved BTK selectivity *versus* kinases outside the TEC family improved clinical toxicity profiles, less profile distinction was evident within the TEC family. Analysis of genetic associations of RA, MS, and SLE biomarkers with TEC family members revealed that BTK and TEC family members may not be drivers of disease. They are, however, mediators of signaling pathways associated with the pathophysiology of autoimmune diseases. BTK in particular may be associated with B cell and myeloid differentiation as well as autoantibody development implicated in immune mediated diseases. Successes in the clinic for treating RA, MS, PV, ITP, and GVHD, but not for SLE and SJ support the concept that BTK plays an important role in mediating pathogenic processes amenable to therapeutic intervention, depending on the disease. Based on the data collected in this study, we propose that current compound characteristics of BTK inhibitor drug candidates for the treatment of autoimmune diseases have achieved the selectivity, safety, and coverage requirements necessary to deliver therapeutic benefit.

## Introduction

Research into the treatment of autoimmune diseases has made great strides toward improving our understanding of disease mechanisms driving pathology. This has led to the development of therapies that go beyond symptomatic treatment and allow for therapeutic interventions that delay or halt disease progression. Biologics targeting proteins such as TNFα for rheumatoid arthritis and IL-17 for psoriasis are prime examples of how knowledge about key nodal points mediating disease mechanisms can lead to therapies that change treatment paradigms for patients. Pharmaceutical research directions are now expanding to include research into areas that seek to understand how immune cells themselves determine the balance of health and disease by changing their functional characteristics and differentiation states in response to cues from their local environment. An example of such a cell type are B cells that are involved in a wide range of activities and play an important role in immunological defense to infection, but yet can also be misdirected to induce autoimmune disease.

Dysregulation of B lymphocytes plays a major role in many of the rheumatic diseases. CD20+ B cell depletion has been shown as an effective therapy for rheumatoid arthritis as well as other autoimmune diseases (1–3). The role of B cells has long been associated with the production of autoantibodies, but B cells and their terminally differentiated plasma cells have been also implicated with the production of proinflammatory cytokines involved in rheumatoid arthritis and other autoimmune diseases (4). Effective therapies targeting B cells in autoimmune pathophysiology remains elusive as shown by studies where B cell depletion therapies have had limited efficacy in diseases such as systemic lupus erythematosus and rheumatoid arthritis, due in part to incomplete B cell depletion and continued production of autoantibodies by other immune cells (5–9).

Bruton’s tyrosine kinase (BTK) is a member of the TEC tyrosine kinase family and is expressed in many hematopoietic cell types, including B cells, mast cells, neutrophils, myeloid cells and osteoclasts. In lymphocyte lineages it is found in B cells, but is not present in T cells or natural killer cells. BTK plays a major role in immune response and is a key mediator of B cell receptor (BCR) and Fc receptor signaling. BCR signaling is also involved with B cell activation and leads to chemotaxis, differentiation, and trafficking (10–12). BTK also renders B cells more sensitive to toll-like receptor signaling events including spontaneous formation of germinal centers, increased IFNγ, IL-6, and IL-1 production, CD80 expression on activated B cells, and anti-nuclear autoantibody induction (13). B cell BTK also mediates other functional responses to activation including maturation, proliferation, antigen presentation, and differentiation into antibody producing plasmablasts and plasma cells. In innate myeloid immune cell types, BTK controls differentiation, cytokine production, phagocytosis, and production of inflammatory mediators (14, 15). In mast cells, BTK plays an important role mediating Fc epsilon receptor signaling to induce chemotactic responses in these cells (16). BTK is also involved in platelet activation *via* the glycoprotein VI receptor (17), osteoclast differentiation in response to receptor activator of NF-κB (RANK) signaling (18), and cell migration in response to certain chemokines.

B cell involvement in disease is often paired with activation of innate immune and stromal cells to cause tissue damage in autoimmune diseases like rheumatoid arthritis (RA) and systemic lupus erythematosus (SLE). In RA patients, B cells may promote disease by several mechanisms including production of autoantibodies and subsequent complement fixation as well as antigen presentation to T cells and subsequent production of inflammatory cytokines and cytotoxic T cell activation. Immune complexes from B cell derived autoantibodies in SLE are an underlying cause of lupus nephritis, depositing in the kidney and activating innate immune cells resulting in tissue damage (19, 20). Support for the role of B cells in disease has been shown in studies where clinical improvement has been established using the anti-CD20 antibody rituximab to deplete mature B cells in rheumatoid arthritis, Systemic lupus and multiple sclerosis (21). Because BTK in B cells plays an important role in B cell activation and differentiation, which in turn mediates the activation of other immune cells such as T cells and myeloid cells that also drive pathology in autoimmune diseases, BTK fits the profile of a key target for therapeutic intervention beyond B cell depletion.

BTK is not without its own challenges for development as a therapeutic. BTK is a member of the TEC family of tyrosine kinases that includes TEC, ITK, BMX, and TXK that share a conserved SH3-SH2-kinase domain making it difficult to achieve specificity when designing small molecule chemical inhibitors (22–25). Ibrutinib was the first approved BTK inhibitor and was found to have broad inhibitory activity across the TEC family and other tyrosine kinases. While it has been approved for B cell related malignancies (Chronic lymphocytic leukemia, Waldenström’s macrogammaglobulinemia, marginal zone lymphoma, and Mantle cell lymphoma), its off-target safety concerns prevent its general development in autoimmune and inflammatory disease indications. Improving the selectivity of BTK inhibitors has been primarily aimed at improving their safety profiles where hemorrhage, atrial fibrillation, and hypertension are common adverse events. By reducing off target inhibition in the second-generation BTK inhibitors, drug developers seek to improve the safety profiles that often result in drug treatment discontinuation. For example, ibrutinib inhibits the activity of 2 major off-targets: EGFR, which can result in severe skin toxicity and TEC family kinases, which decreases their ability to aid in phosphorylation mediated signal transduction (26). Minimizing off-target inhibition to kinases like EGFR and potentially TEC family kinases may be aiding the better toxicity profiles of second generation BTK inhibitors.

In this report, we assessed key topic areas affecting the successful clinical development of BTK inhibitors in autoimmune diseases. We analyzed trial successes and failures for 13 BTK inhibitor drug candidates tested in phase 2 or higher clinical trials representing 7 different autoimmune and 2 different inflammatory disease indications to assess determinants that support further indication development. To reaffirm the biological rationale of using BTK inhibitors, we used pathway enrichment analysis and assessed the relationships between biomarkers of these diseases and the binding to BTK and known off target proteins for these clinical drug candidates. Improvements in selectivity were examined for BTK inhibitors tested in the clinic against some of the most common off target interactions. We further assessed the safety profiles for these BTK inhibitors by reviewing regulatory and scholarly publications.

## Materials and Methods

### Meta-Analysis of Preclinical and Clinical Data

Meta-analysis was performed for BTK inhibitors in clinical development using knowledge databases provided by Clarivate including Cortellis Clinical Trials Intelligence and Web of Science. Cortellis Clinical Trial Intelligence is a comprehensive resource of global clinical trial content from 39 clinical trial registries, press releases, serial publications, corporate and other publications, conference reports, regulatory information and company websites and drug pipelines. In total, this database covered roughly 430K global trials at the time of writing this publication and provided details including trial protocols, status, indications, interventions, and sites as well as linking to the source references. Granular information from this database helped understand all historic and ongoing trials. Certain data and graphics included herein are derived from Clarivate’s Web of Science. ^©^ Copyright Clarivate 2021. All rights reserved. Web of Science^®^ the literature and citation database were used to search 1.7B scholarly references covering journals and conference proceedings indexed back to 1900.

### Biomarker and Pathway Enrichment

Pathway information was obtained from Metabase version (20.4.70300). MetaBase, by Clarivate, is a manually curated knowledge database behind MetaCore, a software suite for pathway and network analysis of high throughput genomics, proteomics, and metabolomics data (27). It contains more than 2.6 million molecular interactions and over 1500 pathway maps which are a collection of canonical signaling, disease, metabolism, and stem cell interactions pathways. The database also has gene-disease associations that are curated by causal facts about gene-disease association, annotated from literature such as linking of SNP with disease risk in a GWAS study or activation of a kinase as a consequence of disease. These gene-disease biomarkers are indexed and annotated with levels of trust based on the evidence that is available from literature sources. For this research, those biomarkers that were significantly association with risk of pathology development, manifestation degree, prognosis of diseases Systemic Lupus Erythematosis (SLE), Rheumatoid arthritis (RA), Multiple sclerosis (MS), Sjogren’s syndrome (SJ) and Pemphigus Vulgaris (PV) were pulled from Metabase.

R programming language was used in conjunction with the MetaBaseR library of scripts to query and run statistical analysis. Pemphigus Vulgaris was excluded from the disease gene interrelationship data set analysis since disease pathway and gene associations for this rare disorder have not been as well studied and thus represented an insufficient dataset for accurate analysis to be performed in conjunction with the other disease datasets.

Pathway map enrichment was run for biomarkers associated with disease. Running the pathway maps enrichment analysis helped understand the biology behind the list of biomarkers associated with the disease by examining the intersection between the biomarker list and a prebuilt map. This defined how enriched the biomarker list is in a particular map. This assessment returned p-value (hypergeometric mean) that state what the likelihood is that the interaction between the biomarker list and a particular map is obtained purely by chance (27).

Pathway map enrichment was also run for the target and off-target list (BTK, ITK, TEC, BMX, TXK, JAK3 and EGFR). Dataframe was created using an R library function to show enrichment p-value for biomarker list and the targets on a single sheet. Green color in protein name columns represents the presence of target and off-target BTK, ITK, TEC, BMX, TXK, JAK2 and EGFR on the listed pathway maps. The p-values generated were calculated using a hypergeometric mean, for the biomarker list on those pathway maps. Microsoft Excel’s conditional formatting was used to color code the p-values with higher range values coded in green, midrange values coded in yellow and lower range values coded in red. The Pathway map list was ranked based on the presence of BTK.

### BTK Inhibitors and IC_50_s

Data on BTK inhibitors were pulled from Cortellis Drug Discovery Intelligence, by Clarivate, a drug research and development pipeline database containing manually curated scientific intelligence for the past 40 years from patents, literature, congresses, conferences, CT.gov, FDA regulations, company press releases and company websites. Pharmacological activity and related IC_50_ values index for the drugs and targets of interest were extracted from the database. Inhibitors with multiple reported IC_50_ values for the same target were averaged together. These values were visually categorized based on the strength of their nanomolar IC_50_s and colored as follows: low nanomolar (<10 nM) in red, low-mid nanomolar (10-100 nM) in orange, high-mid nanomolar (100-999 nM) in yellow, and high nanomolar (≥1000 nM) in green.

### Reported Adverse Events

Data on the reported safety events for these BTK inhibitors were pulled from OFF-X. OFF-X, by bioinfogate, is a translational safety intelligence portal that covers an extensive range of sources including regulatory agencies FDA, EMA & PMDA approval packages (FDA dating back to 1982; EMA since 2015; PMDA since 2019), biomedical literature, Congresses and scientific conferences, major clinical trial registries, real-world evidence studies, Pharmacovigilance databases (i.e FAERS, JADER) and key opinion leaders. These sources cover all adverse events from pre-clinical and clinical studies captured from sources mentioned above. These cover all adverse events reported for the drug used for any indication and were not segmented based on studies for autoimmune disorders.

The scoring is also affected by how widely those individual compounds are studied. The OFF-X Drug Score is powered by Bioinfogate`s unique curated data pool and an advanced rule-based algorithm that screens and weights all the individual pieces of evidence available in the database. The ‘number of alerts’ is defined as the number of data sources (articles, congress abstracts, regulatory agency documents, etc.) reporting a drug related adverse event. The weighting score considers the number of alerts, type of primary source, type of study, and evidence based on type of reference and development phase. The score is adjusted to allow the comparison of drugs at different stages of development. This includes a factor that takes into account the overall number of publications available in OFF-X for each drug. Very high evidence on the heat map is represented in red, which means there is confirmatory evidence from regulatory bodies supporting a drug-adverse event association and that there are multiple reports from sources supporting it. The darker orange color represents high evidence when at least one regulatory body has reported evidence supporting a Drug-Adverse Event association or, in the absence of communications from regulatory bodies, the evidence is supported by a significant number of publications from other data sources. Lighter orange color represents medium evidence when the evidence supporting a Drug-Adverse Event association has been reported in several data sources or is under review by a regulatory agency. Yellow color represents low evidence when some preliminary evidence supporting a Drug-Adverse Event association has been reported either in humans or during preclinical studies. Grey represents target class evidence only when only class alerts are available for a given adverse event and the drug of interest was not explicitly mentioned in the data source(s). This score label also includes alerts associated to target genetic studies or mutations. Blue represents where there is evidence that refutes a drug adverse event association. White indicates that no evidence is currently available.

## Results

### BTK Inhibitors in Clinical Trials for Autoimmune Indications

BTK inhibitors that have been or currently are in clinical trials for the treatment of autoimmune and inflammatory immune-mediated diseases were assessed for clinical trial stages of development, disease indications, clinical results, nature of the inhibitor (covalent *versus* noncovalent), and development status. Thirteen compounds targeting BTK in 9 indications having attained clinical trial status of phase 2 or beyond were found that encompass RA, SLE, MS, PV, SJ chronic spontaneous urticaria (CSU), idiopathic thrombocytopenia (ITP), graft *versus* host disease (GVHD) disease, and asthma indications (**Table 1**). Eleven of these BTK inhibitor clinical drug candidates are covalent alkylating agents, forming an irreversible bond with cysteine 481 in the ATP binding pocket. Of the 13 BTK inhibitors tested in the clinic, only 2 have noncovalent inhibition mechanisms of action, BMS-986142 and fenebrutinib.

**Table 1:** Clinical BTK inhibitors in phase 2 or higher clinical trials. BTK inhibitors are listed by registered names if available.

Drug	Covalent	RA	SLE	MS	SJ	PV	CSU	ITP	GVHD	Asthma	Trial No./Indication/Status/Trial length
BMS-986142	N	2(-)									NCT02638948/RA/completed/12 wks
					2						NCT02843659/SJ/terminated/12 wks
Branebrutinib	Y	2									NCT04186871/RA/ongoing/12 wks
			2								NCT04186871/SLE/ongoing/24 wks
					2(-)						NCT04186871/SJ/ongoing/24 wks
Acalabrutinib	Y	2									NCT02387762/RA/completed/4 wks
Elsubrutinib	Y	2									NCT03682705/RA/completed/12 wks
			2								NCT03978520/SLE/recruiting/24 wks
Evobrutinib	Y	2(-)									NCT02784106/RA/completed/12 wks
			2(-)								NCT02975336/SLE/completed/52 wks
				2(+) 3							NCT02975349/MS/ongoing/48 wks
Fenebrutinib	N	2(+)									NCT03233230/RA/completed/12 wks
			2(-)								NCT02908100/SLE/completed/48 wks
				2							NCT04586023/MS/ongoing/96 wks
							2(-)				NCT03693625/CSU/terminated/24 wks
Ibrutinib	Y								(+)		Approved. Multiple ongoing expansion trials
Poseltinib	Y	2(-)									NCT02628028/RA/terminated/4 wks (part A)
Remibrutinib	Y				2						NCT04035668: SJ/ongoing/24 wks
							2				NCT03926611/CSU/ongoing/16 wks
										2(-)	NCT03944707/Asthma/terminated/12 wks
Rilzabrutinib	Y					2(+) 3(-)					NCT02704429/PV/completed/24 wks
								2(+) 3			NCT04562766/ITP/ongoing/24 wks
Spebrutinib	Y	2(-)									NCT01975610/RA/completed/4 wks
Tirabrutinib	Y	1									NCT02626026/RA/completed/4 wks
					2(-)						NCT03100942/Sj/completed/24 wks
Tolebrutinib	Y			2(+)							NCT03889639/MS/completed/16 wks
				3							NCT04410978/MS/ongoing/36 months

Indications being tested for each BTK inhibitor have the associated phase status number and positive (+) or negative (-) results indicated if reported. The assigned clinical trial number/indication/trial status/and length of trial is referenced for each indication. If more than one trial exists for a specific BTK inhibitor in an indication, only the clinical trial supporting the main efficacy of the BTK inhibitor is listed. N, no; Y, yes; Covalent, BTK inhibitor forms a covalent bond with BTK; RA, rheumatoid arthritis; SLE, systemic lupus erythematosus; MS, multiple sclerosis; SJ, Sjogren’s disease; PV, pemphigus vulgaris; CSU, chronic spontaneous urticaria; ITP, idiopathic thrombocytopenic purpura; GVHD, graft versus host disease.

RA represented the most targeted indication for second generation BTK inhibitors with 9 of the 13 clinical compounds having been tested in the clinic with mixed results. Five of these drug candidates have reported clinical results, with four failing to show significant improvement in RA (BMS-986142, branebrutinib, polsetinib, and spebrutinib) and one reporting positive improvement (fenebrutinib). Of the four failed trials reported in RA, branebrutinib, polsetinib, and spebrutinib drug candidates are covalent BTK inhibitors that were tested over periods of 12 weeks, 12 weeks, and 4 weeks, respectively and one was noncovalent (BMS-986142) tested over a 4 week trial period. The successful fenebrutinib is a noncovalent inhibitor tested over a 12 week period at 50 and 150 mg once daily and 200 mg twice daily.

SLE was the next most pursued indication for BTK inhibitors with 4 drug candidates having been tested in clinical trials (branebrutinib, eslubrutinib, evobrutinib, and fenebrutinib). Of the two BTK inhibitors reporting results (evobrutinib and fenebrutinib), no significant impact on disease was reported. Evobrutinib is a covalent inhibitor that was given 150 mg QD and 200 mg BID for 52 weeks and assessed by SRI-4 and BICLA (28). Fenebrutinib is a noncovalent inhibitor that was given 25 or 75 mg once daily or 50 mg twice daily over a 48 week period and assessed by the SLE-Responder Index-4 and 6 (29).

Multiple sclerosis was tested in phase 2 trials with two BTK inhibitors (evobrutinib and tolebrutinib), both of which demonstrated positive disease impact on this neurodegenerative disorder and are covalent modifiers of BTK. Evobrutinib reported a reduction in disease relapse rate at 75 mg twice daily for 108 weeks in the open label phase 2 study is currently in a 108 week phase 3 trial. Data from a 12 week phase 2b trial of tolebrutinib demonstrated efficacy in reducing lesions and relapse rates in MS subjects.

Sjogren’s disease was tested with four BTK inhibitors (BMS-986142, branebrutinib, remibrutinib, and tirabrutinib). BMS-986142 is a the only noncovalent BTK inhibitor of the 4 drug candidates and were given to subjects over a 24 week period. Two negative results were reported (branebrutinib and tirabrutinib), while the other two have not reported results (remibrutinib and BMS-986142). BMS-986142 is no longer listed as being actively pursued on the company website.

Three additional autoimmune diseases, PV, ITP, CSU as well as two inflammatory immune-mediated diseases, GVHD and asthma, were diseases where two or fewer BTK inhibitors in clinical trials were tested. PV was evaluated with the BTK inhibitor rilzabrutinib and demonstrated significant improvement of skin lesions, leading to its progression to phase 3 clinical studies that failed to meet the higher standard primary endpoint of complete absence of new and established skin lesions with minimal steroid use. ITP was being tested with rilzabrutinib and reported positive interim results. CSU was tested with fenibrutinib and reported negative results, while remibrutinb data has not been reported. Ibrutinib was the only BTK inhibitor tested in GVHD and has been approved by the FDA for this indication, supported in part by a phase 2 clinical trial where 69% of transplant patients achieved an overall response to drug (31% complete response; 38% partial response) and 55% maintained a response of at least 44 weeks (30). Additional improvements in patients with sclerosis (61%) and reductions in steroid use (64%) were also observed.

### BTK Inhibitors Have Similar Disease Model Activity

Autoimmune animal models used to profile the 13 clinical candidates were tabulated according to disease type (**Table 2**). The table shows there is a limited range of *in vivo* models used to profile BTK inhibitors. When preclinical disease models were reported, all disclosed positive activity. Lack of reported data cannot be interpreted as having positive or negative results. The most common *in vivo* model employed BTK inhibitor characterization was in mouse and rat arthritis models and was reported by 9 of the 13 clinical BTK inhibitors. Acalabrutinib did not report CIA model tests, but did proceed to a 4 week trial for RA without reporting results. Tolebrutinib was the only BTK inhibitor not reporting CIA model tests and not pursing RA as an indication. Three other BTK inhibitors (ibrutinib, remibrutinib, and rilzabrutinib) reported positive CIA model results, but did not purse an RA indication. Eight BTK inhibitors were profiled in SLE models, 3 in MS experimental encephalomyelitis (EAE) models, 1 in a model of ITP, and 1 in a model of GVHD. The most commonly used animal models paralleled the number of clinical drug candidates in each correlated human disease condition (arthritis>Lupus>MS) and with few exceptions, each clinical trial indication pursued was also tested in the corresponding animal model. While Sjogren’s disease was a clinical focus for 4 BTK inhibitors (BMS-986142, branebrutinib, remibrutinib, tirabrutinib), no pre-clinical data was reported.

**Table 2:** BTK inhibitors tested in preclinical animal disease models. BTK inhibitors tested in animal models of disease are listed if they have been reported.

BTK Inhibitor	Other Names	RA Models	SLE Models	MS Model	ITP Model	References
BMS-986142	BMS-986142	CIA mouse CAIA mouse	NZB/W			(31, 32)
Branebrutinib	BMS-986195	CIA mouse CAIA mouse	NZB/W			(33, 34)
Acalabrutinib	calquence ACP-196	CIA mouse	MRL/lpr			(35)
Elsubrutinib	ABBV-105	CIA rat	NZB/W MRL/lpr			(36, 37)
Evobrutinib	M2951	CIA mouse CIA rat	IFN-alpha NZB/W	MOG EAE		(38–41)
	MSC-2364447					
Fenebrutinib	GDC-0853 RG7845	CIA rat		PLP EAE		(42, 43)
Ibrutinib	imbruvica, PCI-32765	CIA mouse CIA rat	MRL/lpr B6.Sle1 B6.Sle1.Sle3			(35, 44–47)
Poseltinib	LLY-333764	CIA mouse	NZB/W MRL/lpr			(48–50)
	HM-71224					
Remibrutinib	LOU064	CIA rat				(51, 52)
Rilzabrutinib	PRN1008	CIA mouse CIA rat			Mouse anti-CD41	(53, 54)
Spebrutinib	CC-292	CIA mouse	NZB/W MRL/lpr			(55–57)
	AVL-292					
Tirabrutinib	GSK-4059 ONO-4059	CIA mouse CIA rat sRANKL	NZBWF1			(58–60)
Tolebrutinib	SAR442168 PRN2246			MOG EAE		(61)

If more than one type of model was performed it was listed only if a different animal strain, species, or major mechanism of action was employed. CIA, collagen induced arthritis; CAIA, collagen antibody induced arthritis; sRANKL, soluble receptor activator for nuclear factor κB ligand; MOG EAE, myelin oligodendrocyte glycoprotein experimental autoimmune encephalomyelitis; PLP EAE, proteolipoprotein experimental autoimmune encephalomyelitis; IFN-alpha, interferon-alpha. Remaining abbreviations refer to animal strains used.

### BTK Signaling Pathways Correlate Differentially With Disease Gene Associations

BTK and other TEC family genes were not found to have genetic evidence linking them with the diseases tested in the clinic (RA, MS, SLE, and Sjögren’s), but signal pathway analyses show their involvement in relevant disease processes (**Table 3** and **Supplementary Table 1**).

**Table 3:** Pathway map enrichment with disease biomarkers.

Pathway Map Name	BTK	TEC	ITK	BMX	TXK	JAK3	EGFR	SLE	RA	MS	SJ
								p-value	p-value	p-value	p-value
Chemotaxis_CXCL12/CXCR4-induced chemotaxis of immune cells								8.28E-08	2.44E-05	3.01E-03	2.41E-03
Role of tumor-infiltrating B cells in anti-tumor immunity								3.33E-25	4.25E-21	1.55E-16	7.66E-29
Immune response_NF-AT in immune response								5.40E-12	3.25E-07	1.34E-03	3.95E-03
Immune response_IL-6 signaling pathway *via* MEK/ERK and PI3K/AKT cascades								2.24E-08	1.16E-07	5.01E-04	3.42E-04
Immune response_PIP3 signaling in B lymphocytes								1.42E-02	3.68E-02	NA	NA
Role of B cells in SLE								1.39E-28	9.93E-30	2.17E-18	2.97E-26
SLE genetic marker-specific pathways in B cells								2.51E-24	2.66E-14	4.12E-11	6.54E-10
B cell signaling in hematological malignancies								1.55E-18	1.64E-13	8.03E-07	1.15E-08
Immune response_Fc epsilon RI pathway: Lyn-mediated cytokine production								4.65E-18	2.04E-09	5.00E-05	3.77E-08
IgE- and MGF-induced Lyn-mediated production of cytokines and arachidonic acid metabolites in lung mast cells in asthma								1.47E-17	5.94E-09	8.95E-06	1.05E-04
Immune response_IL-5 signaling *via* PI3K, MAPK and NF-κB								5.41E-12	9.14E-12	1.71E-04	4.18E-04
Immune response_B cell antigen receptor (BCR) pathway								1.37E-10	6.13E-06	NA	1.06E-04
Immune response_BAFF-induced canonical NF-κB signaling								3.88E-07	1.46E-08	1.73E-03	3.98E-07
Cell adhesion_Integrin inside-out signaling in neutrophils								1.83E-05	5.68E-05	2.45E-03	4.61E-04
Immune response_TLR3 and TLR4 induced TICAM1-specific signaling pathway								2.31E-05	4.89E-07	2.92E-02	4.38E-05
Proinflammatory mediators release and arachidonic acid metabolites production in basophils in asthma								1.21E-03	4.03E-05	1.02E-02	2.72E-02
Blood coagulation_GPVI-dependent platelet activation								2.53E-03	3.35E-03	NA	NA
G-protein signaling_G-Protein alpha-q signaling cascades								3.78E-03	3.38E-02	NA	NA
Production of reactive oxygen species and arachidonic acid metabolites by neutrophils in asthma								1.50E-02	1.19E-03	9.73E-03	1.58E-02

Enrichment results generated using pathway map enrichment analysis in MetaCore. Left side shows the presence of the target/off-target on the map and the right corner shows the p-value for the biomarker list colored as: Highly significant in red, mid value in yellow and low significance in green.

There was little overlap of BTK with the other TEC family members in disease associated pathways. Of the 19 identified disease associated signaling pathways where BTK was involved, two pathways also contained ITK (“Chemotaxis_CXCL12/CXCR4-induced chemotaxis of immune cells”, “Immune response_NF-AT in immune response”) and two pathways contained TEC (“Immune response_IL-6 signaling pathway *via* MEK/ERK and PI3K/AKT cascades”, “Immune response_PIP3 signaling in B lymphocytes”).

**Figure 1:**
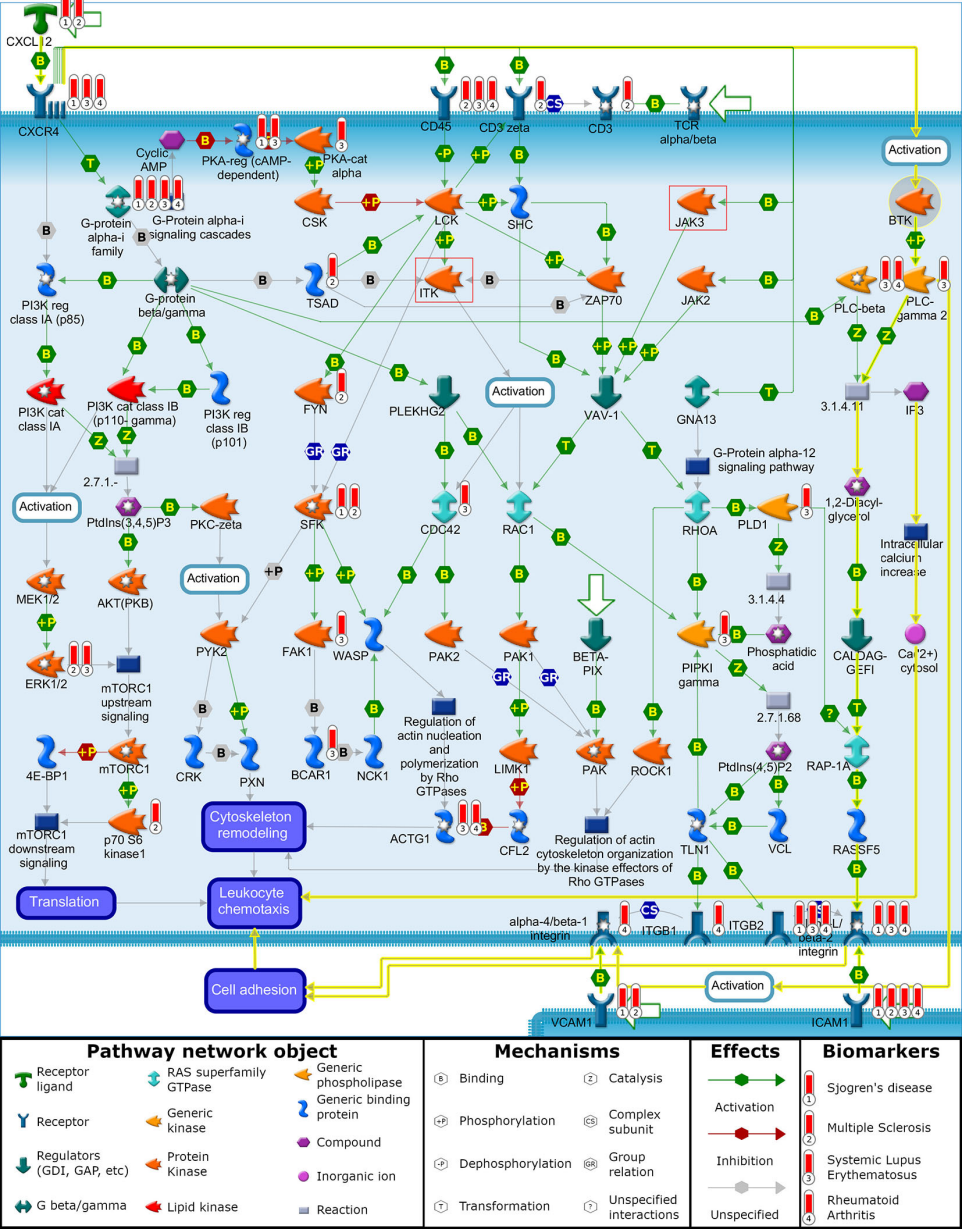
“Chemotaxis_CXCL12/CXCR4-induced chemotaxis of immune cells”. Illustration generated with MetaCore pathway analysis tool indicating the involvement of BTK and disease biomarkers to induce chemotactic response in immune cells leading to cell adhesion and cell migration. Data overlaid onto the pathway map are represented as thermometers which typically would indicate the level of over/under expression of a gene. Thermometer 1 represents SJ biomarkers, thermometer 2 represents MS biomarkers, thermometer 3 represents SLE biomarkers, and thermometer 4 represents RA biomarkers. For demonstration purposes, each of these biomarkers was given a value of 1.

The first pathway that BTK and ITK are involved in is the “chemotaxis_CXCL12/CXCR4-induced chemotaxis of immune cells”. To understand the mechanistic relationship between the targets and biomarkers, a visualization (**Figure 1**) of this pathway map was overlaid with the biomarker list and target/off-target list on top of this map for further analysis. This pathway also has JAK3 and showed a statistically significant p-value for the SLE biomarker list. In this map, CXCL12 is indicated as a disease biomarker and initiates signaling *via* its CXCR4 receptor to induce chemotactic responses in diverse immune cell types. Upon activation, CXCR4 couples to G-protein alpha-i family, G-protein beta/gamma and G-protein alpha-13 to activate phosphatidylinositol 3-kinase (PI3K)-, Rho GTPases- and Phospholipase C (PLC)-dependent pathways that regulate protein synthesis, cytoskeleton remodeling and integrin-dependent cell adhesion. In addition, CXCL12/CXCR4 transactivates the TCR alpha/beta-CD3 complex leading to integrin-mediated cell adhesion and directional cell migration. Chemotaxis is also involved in the “Role of tumor-infiltrating B cells in anti-tumor immunity” pathway map and includes BTK and JAK3, but does not include ITK. This pathway map is associated with all four diseases listed (SLE, RA, MS and SJ).

The second pathway, “Immune response NF-AT in immune response” (**Table 3**), involved BTK and ITK and showed significant p-values in the SLE and RA biomarker list. But, unlike the first pathway map, “chemotaxis_CXCL12/CXCR4-induced chemotaxis of immune cells”, this pathway does not contain JAK3 in the signaling cascade. This pathway map does show the role of nuclear factors of activated T cells (NF-AT) transcription factors in initiation and coordination of the immune response in immunocytes including T- and B cells, mast cells, basophils and natural killer cells.

The two BTK pathways that included TEC, the “Immune response_IL-6 signaling pathway *via* MEK/ERK and PI3K/AKT cascades” and “Immune response_PIP3 signaling in B lymphocytes” have less correlation with the disease biomarkers. The “Immune response_IL-6 signaling pathway *via* MEK/ERK and PI3K/AKT cascades” pathway map was only correlated with SLE and RA. The second BTK and TEC shared pathway map “Immune response_PIP3 signaling in B lymphocytes” had poor p-values for SLE and RA and no association with MS and SJ.

### Second Generation BTK Inhibitors Are More Selective, Though Variable Within the TEC Family

Observing the reported IC_50_ values across seven kinases being compared, several trends are seen (**Table 4**). BTK is the main therapeutic target for these compounds and each has a reported IC_50_ in the low to mid nanomolar range. The range of BTK inhibition varies from elsubrutinib in the hundreds of nanomolar range to BMS-986142, branebrutinib, and tolebrutinib in the sub nanomolar range.

**Table 4:** IC_50_ values were collected from data provided in Cortellis Drug Discovery Intelligence.

Name (synonyms)	BTK	TEC	ITK	BMX	TXK	JAK3	EGFR
Branebrutinib (BMS-986195)	0.1 *	0.9	100.0	1.5	5.0	No data	2,000.0
BMS-986142	0.7 *	21.2 *	507.5 *	85.0 *	87.5 *	1,000.0	1,000.0
Tolebrutinib (SAR-442168, PRN2246)	0.9 *	1.0	365.0	1.1	1.7	1425.0 *	4.1
Poseltinib (LY-3337641, HM-71224)	2.3 *	4.5	103.0	0.6	4.6	18.8 *	26.0 *
Ibrutinib (imbruvica, PCI-32765)	2.4 *	37.2 *	91.9 *	1.2 *	4.5 *	51.8 *	30.2 *
Fenebrutinib (GDC-0853)	5.3 *	>1000	>1000	351.0	>1000	>1000	>1000
Remibrutinib (LOU064)	5.5 *	No data	No data	No data	No data	No data	No data
Tirabrutinib (GSK-4059, ONO-4059)	7.8 *	29.0 *	>1000	21.7 *	203.5 *	>1000	>1000
Acalabrutinib (calaquence, ACP-196)	10.4 *	142.1 *	>1000	91.6 *	427.8 *	>1000	>1000
Spebrutinib (CC-292)	15.4 *	10.2 *	674.7 *	6.4 *	93.7 *	75.3 *	436.0 *
Rilzabrutinib (PRN1008)	33.4 *	0.8 *	440.0	1.0 *	1.2 *	>5000	520.0
Evobrutinib (M2951)	47.7 *	≤ 100	≤ 10000	> 10	>100	10,000.0	10,000.0
Elsubrutinib (ABBV-105)	175.0 *	23,400.0	32,400.0	No data	9,180.0	8,640.0	14,400.0

Instances where multiple values were averaged together and noted with a *. For analysis, these values were binned together based on the strength of their nanomolar IC_50_s and colored as follows: low nanomolar (<10 nM) in red, low-mid nanomolar (10-100 nM) in orange, high-mid nanomolar (100-1000 nM) in yellow, and high nanomolar (>1000 nM) in green.

Within the TEC kinases, we compared TEC, ITK, BMX, and TXK kinase inhibition. BMX inhibition for most of these inhibitors was in the low to low-mid nanomolar range. Overall, the inhibition of TEC, BMX, and TXK are still in the realm of being clinically relevant with IC_50_ values in the low to high-mid nanomolar range for these covalent inhibitors. The two exceptions to this are fenebrutinib and elsubrutinib which are both non-covalent inhibitors of BTK. The inhibition of ITK was weaker then ibrutinib for all the drugs examined.

Outside of the TEC kinase family, we examined the off-target selectivity of these BTK inhibitors against JAK3 and EGFR. For the majority of the second generation BTK inhibitors, inhibition of JAK3 and EGFR was in the high micromolar range. Only poseltinib, ibrutinib, and spebrutinib have reported IC_50_s for JAK3 and EGFR in the clinically relevant low-mid to high-mid nanomolar range. Rilzabrutinib and tolebrutinib had EGFR IC_50_ values in the high-mid and low nanomolar range, respectively.

### OFF-X Safety Alerts Show Similar Safety Improvements

Of the BTK inhibitors evaluated in this paper, most are still too early in development to have a substantial body of literature evidence to report adverse events on. The “numbers of alerts” reported in OFF-X is defined as the number of data sources reporting a given drug related adverse event. As detailed in the materials and methods section, the drug score estimates the strength of evidence supporting a given drug related adverse event association based on the safety alerts available in OFF-X. Of the 13 BTK inhibitors being studied, safety information for elsubrutinib is not yet available in the database. For the remaining 12 BTK inhibitors with reported safety concerns, there were a total of 5272 reported alerts across 853 adverse events (**Table 5** and **Supplementary Table 2**). Three BTK inhibitors had enough safety alerts evidence to receive a very high drug score by OFF-X: ibrutinib (3136 total alerts), acalabrutinib (1383 total alerts), and tirabrutinib (493 total alerts). Three BTK inhibitors had enough safety alerts reported to receive medium evidence drug scores by OFF-X: evobrutinib (123 total alerts), fenebrutinib (52 total alerts), and rilzabrutinib (18 total alerts). The other 6 BTK inhibitors have not accumulated enough evidence from literature to achieve a drug score above low evidence.

**Table 5:** OFF-X Comparative Drug Safety Evidence showing safety findings for BTK inhibitor.

ADVERSE EVENT	ALERT TYPE				ACALABRUTINIB	BMS-986142	BRANEBRUTINIB	EVOBRUTINIB	FENEBRUTINIB	IBRUTINIB	POSELTINIB	REMIBRUTINIB	RILZABRUTINIB	SPEBRUTINIB	TIRABRUTINIB	TOLEBRUTINIB
	CLASS ALERT		DRUG ALERT													
	# of alerts	# of drugs	# of alerts	# of drugs	# of alerts											
All Adverse Events (853)	26	4	5096	12	1383	10	11	123	52	3136	23	3	18	14	493	6
Atrial fibrillation	2	1	211	3	50	0	0	0	0	163	0	0	0	0	2	0
Diarrhoea	2	1	171	8	58	1	0	2	2	111	0	0	4	1	11	0
Haemorrhage	2	1	159	3	55	0	0	0	0	110	0	0	0	0	4	0
Neutropenia	1	1	153	5	49	0	0	1	0	99	0	0	0	1	12	0
Hypertension	1	1	135	4	32	0	0	1	0	104	0	0	0	0	1	0
Pneumonia	0		129	7	45	0	0	1	1	78	1	0	0	1	4	0
Anaemia	0		126	6	47	0	0	0	3	73	1	0	0	1	9	0
Infection	1	1	110	6	29	0	0	3	1	79	1	0	0	0	2	0
Thrombocytopenia	0		108	5	27	0	0	0	2	77	0	0	0	1	9	0
Fatigue	0		107	6	36	0	0	1	3	70	0	0	3	0	2	0
Nausea	0		99	9	33	1	1	6	4	49	0	0	4	1	7	0
Headache	0		95	10	58	1	1	3	3	28	1	0	1	0	1	1
Contusion	0		94	4	36	0	0	0	1	55	0	0	0	0	7	0
Arthralgia	0		85	6	21	0	0	2	1	62	1	0	0	0	3	0
Rash	0		79	6	14	0	1	0	1	58	1	0	0	0	9	0
Upper respiratory tract infection	0		69	8	22	1	1	4	2	36	0	0	0	0	3	1
Death	0		59	4	18	0	0	0	1	35	0	0	0	0	6	0
Pyrexia	0		57	4	14	0	0	0	1	39	0	0	0	0	5	0
Cough	0		56	6	22	1	0	0	2	27	0	0	0	1	4	0
Myalgia	0		49	4	18	0	1	0	0	32	0	0	0	0	1	0

The number on the table is the count of alerts for each drug associated with that adverse event. The background color represents the drug score linking the strength of evidence between each adverse event and the drug: red is very high evidence, orange is high evidence, lighter orange color is Medium Evidence, yellow is low evidence, grey is target/class evidence only, and white indicates no evidence is available.

Further analysis will focus on the compounds with enough alerts to obtain drug scores with very high evidence (acalabrutinib, ibrutinib, and tirabrutinib) in the top 20 reported adverse events (**Table 5**). Diarrhea, neutropenia, anemia, thrombocytopenia, and rash all have high or very high evidence scores in OFF-X. Hemorrhage, hypertension, pneumonia, infection, fatigue, nausea, contusion, arthralgia, and upper respiratory infection all have very high evidence for acalabrutinib and ibrutinib, but tirabritinib only reached low to medium drug score evidence for these adverse events. It is important to note that tirabrutinib (493 safety alerts) has only been launched in Japan last year and has had less time to accumulate as many reported events as ibrutinib, which was launched in 2013 (3136 safety alerts) or acalabrutinib, which was launched in 2017 (1383 safety alerts). Atrial fibrillation has the highest count of alerts in ibrutinib with 163 out of 3136 alerts and a very high evidence score as compared to acalabrutinib with 50 out of 1383 alerts and a lower high evidence score for the same adverse event. Tirabrutinib has 2 out of 493 alerts and ranked as an OFF-X drug candidate with a low evidence score. Headache was reported for acalabrutinib in 58 alerts and has a very high drug score, ibrutinib has 28 alerts with a high drug score, and tirabrutinib has 1 alert with a medium drug score. High drug scores for death were attributed to acalabrutinib (18 alerts) and ibrutinib (35 alerts) while tirabrutinib (6 alerts) has a medium drug score. Pyrexia has a very high drug score in ibrutinib (39 alerts) while acalabrutinib and tirabrutinib only have medium drug scores (14 and 5 alerts respectively). Cough has a high drug score for ibrutinib (27 alerts) while acalbrutinib and tirabrutinib have medium drug scores (22 and 4 alerts respectively). Myalgia has a high drug score for acalabrutinib (18 alerts) while ibrutinib and tirabrutinib have medium drug scores (32 and 1 alert respectively).

## Discussion

### Clinical Study Paradigms and Covalent BTK Inhibition

The discovery of the essential roles that BTK plays in B cell development, trafficking, and antibody production has opened a new area for treating autoimmune disorders. In surveying clinical trials that have been or currently are in the testing phase for BTK inhibitors, we found that indication selection has been biased towards the treatment of the rheumatic diseases RA and SLE, with a few trials also conducted for the treatment of MS, PV, SJ, CSU, IP, GVHD, and asthma. We also found advancements in drug selectivity and the inclusion of covalent bond formation have led to improvements in effective BTK inhibition coverage and clinical safety parameters in subjects treated with this new generation of BTK inhibitors. Therapeutic efficacy has, however, been mixed with some reports showing encouraging data on disease improvement that may be dependent on indication and in the case of RA, also dependent on length of trial duration. While the limited clinical data currently available precludes thorough assessment of the different BTK inhibitors for correlations of efficacy to potency, selectivity, treatment paradigms, and exposure variables, these associations should become more clear as trials in immunological diseases progress.

In surveying the drug candidates reaching phase 2 or later, we found that the most distinguishing characteristic of the new group of BTK inhibitors was the ability to covalently bind to the enzyme and permanently inhibit activity. This suicide inhibitor approach to enzyme inhibition is intended to enhance good drug profile characteristics in three areas: selectivity, near complete enzyme inhibition, and a longer time course of effect (effective drug coverage/exposure). To that end, covalent modification of cysteine 481 was the focus of the majority of the clinically tested BTK inhibitors in autoimmune disease indications, representing 11 of the 13 drug candidates (**Table 1**).

As indicated, the first improvement area of the new generation of BTK inhibitors was in selectivity and all but two of these drug candidates employed the use of covalent bond formation to reduce off-target binding to other protein kinases. These covalent inhibitors were designed to bind to the ATP binding site of BTK and use a reactive chemical “warhead” in their structure to covalently modify cysteine 481 of the enzyme. The design of these warhead compounds is intended to provide enough selective noncovalent binding interactions between the inhibitors and BTK to limit widespread off-target kinase inhibition, orient the molecule’s proximity to the binding pocket cysteine, and to give appropriate time for covalent bond formation to occur. The result is that these covalent inhibitors have had success at limiting strong inhibition to only kinases that have cysteine in their ATP binding pocket, including the 5 TEC family kinases (BTK, BMX, TEC, ITK and TXK) and 6 other kinases (EGFR, ERBB2, ERBB4, JAK3, BLK and MKK7). While an improvement in selectivity for BTK inhibition in the larger general family of protein kinases was achieved near uniformly with this targeted “warhead” paradigm, achieving selectivity within the TEC family and ATP cysteine pocket related kinases was variable (**Table 4**). The most nonselective drug first developed with this covalent mechanism, ibrutinib, has a high off target toxicity profile (**Table 5**) and so its use has been limited to mostly oncology indications (62). This broad off-target activity has, however, been shown to be useful in treating graft *versus* host disease that develops in cancer treatments and has been approved by the FDA for this indication (63). The remaining BTK inhibitors have shown much improved selectivity due in part to the use of cysteine 481 modification. It should be noted, however, that one drug candidate (fenebrutinib) has shown near complete BTK selectivity within the TEC family without the use of cysteine 481 covalent modification (**Tables 1**, **4**).

The second area of enhanced drug characteristics conferred by covalent BTK inhibitors is the potential to maximize inhibition at a given drug concentration. While binding affinity and dissociation rates determine KD affinity constants, it is the addition of the suicide inhibition rate constant of modifying cysteine 481 that effectively increases the potency of the compound (reduces the observed IC_50_) and may be a more accurate measure distinguishing inhibition among covalent BTK inhibitors (64). Once covalently bound, the enzyme is removed permanently from contributing functional activity in the cell. With the stoichiometric excess of drug to enzyme ratio that occurs with typical treatment doses, covalent binding of BTK inhibitors serves to increase potency at a given concentration of drug and makes it possible to show near complete inhibition of BTK function at physiologically attainable drug concentrations.

The third contribution to enhancement of good BTK inhibitor drug characteristics imparted by covalent inhibition occurs by extending the time of inhibition of BTK beyond clearance of the drug from circulation that normally reduces drug exposure. For covalent BTK inhibitors that permanently inactivate the enzyme, recovery of activity is a slower process determined principally by synthesis of new BTK in the cell. In a mouse study, recovery of uninhibited BTK after spebrutinib treatment was reported to be 43% by 24h and 71% by 48h post after a single 50 mg/kg administration of this drug candidate (55). The persistence of BTK inhibition occurred long after complete clearance (8h) of circulating spebrutinib in the plasma of these mice. Similar separation of circulating drug levels and inhibitory capacity of spebrutinib was observed in a healthy volunteer study where peak BTK inhibition of 98% covalent drug occupancy was achieved despite a rapid clearance half-life in plasma of 1.9 h. This was followed by an extended period of BTK inhibition as shown by a slow 25% free BTK recovery at 24h and 50% between 48-72h (55). Slow recovery of BTK activity due to new synthesis of enzyme in human subjects was also seen after acalabrutinib treatment in patients with chronic lymphocytic leukemia, where a short half-life of plasma concentrations of 1h was observed (65), yet only a 25.6% of free BTK was recovered by 48h post last dose (66). Given that BTK resynthesis rates among individuals can vary, as was observed in the Acalabrutinib trial (3.6% to 31.4%), and that generally the safety profiles are improved with second generation BTK inhibitors (**Table 5**) due in part to enhanced selectivity (**Table 4**) and rapid drug clearance, twice daily dosing at high drug coverage levels has been the method of choice for clinical application of BTK inhibitors.

It is unclear what threshold of BTK inhibition is required for clinical efficacy in treating autoimmune diseases and so the majority of these covalent inhibitors have included twice daily dosing regimens that achieve near complete inhibition of BTK activity. For example, near complete occupancy was observed in clinical studies with BID dosing reaching 96% for acalabrutinib (66) and 88% for spebrutinib in clinical trials of RA patients (67). These studies also demonstrated that extended periods of near complete BTK inhibition are relatively safe as reported by few adverse events. The safety concern for near complete inhibition of BTK was the potential that such inhibitors may result in severe reduction in B cells and other immune cell alterations as was observed in mice that bear the X-linked immunodeficiency (xid) mutation in BTK (68–70) and in humans that bear the X-linked agammaglobulinaemia (XLA) human immunodeficiency mutation in BTK (71, 72). It appears, however, that the low levels of BTK activity remaining after near complete BTK inhibition by covalent cysteine modifying drug candidates is sufficient to support enough immunological function to limit compound toxicity profiles and yet decouple BTK activity from disease inducing autoimmune mechanisms (**Tables 1** and **5**) (73). Since near complete inhibition of BTK appears to be well tolerated in autoimmune clinical trials and trials are just completing early proof of concept studies, little investigation has been done yet as to what the levels of minimal effective doses required for clinical efficacy are with the various BTK inhibitors, what that efficacy relationship is relative to the percent of BTK inhibition attained, and what dosing schedules and recovery rates of BTK activity are allowable without disease worsening.

### BTK Clinical Efficacy in Rheumatoid Arthritis

While threshold coverage appears to be sufficient to observe clinical efficacy for BTK inhibitors, it is not clear what the actual immune mechanisms impacted are that achieve this efficacy. A look at RA mechanisms and subtypes as well as trial methodology may help to understand the impact of BTK inhibition. Based on the evidence from preclinical and clinical studies, a hypothesis can be proposed that the impact of BTK inhibition on RA disease pathology may be more by altering B cell development affecting subsequent immune processes that are dependent on B cell differentiation than by directly affecting inflammatory activity. In this scenario, disease impact of BTK inhibitors would take time to occur relative to the rapid effects observed for current drugs targeting ongoing inflammation (e.g. steroids and anti-TNFα). Early clinical studies of BTK inhibitors focused heavily on RA as their first clinical indication with 9 of the 13 BTK inhibitors having been tested, yet only 1 (fenebrutinib) reported positive data [(74); **Table 1**]. In the latter fenebrutinib study, a 50% improvement criteria (ACR50) was achieved at week 12 and comparable in the fenebrutinib 150 mg once daily dose group (28%) and the 200 mg twice daily dose group (35%) *versus* the placebo group (15%). In addition, efficacy was comparable to treatment with the anti-TNFα antibody, Adalimumab (36%).

In contrast to the poor clinical success rate of BTK inhibitors for treating RA, nearly all have reported positive results in animal models of arthritis (**Table 2**). At first glance, it might be concluded that mouse arthritis models are not predictive of the human RA condition. Conversely, it is possible that this mouse model/human disease prediction discrepancy is due to the testing of BTK inhibitors prophylactically in mice before pathological B cell differentiation effects occur rather than therapeutic dose designs (post disease onset).

The human RA condition is already well developed by the time treatment occurs and is an overlay of beginning and end stage differentiation and inflammatory processes occurring concomitantly and cyclically, which may affect trial time requirements for determining clinical efficacy for BTK inhibitors. In this scenario, phase 2 clinical trials for RA methods targeting inflammation with steroids and anti-TNFα biologics can be observed in a very short time, often within 4 weeks that typical phase 2 RA trials are performed with these drugs. Four BTK drug candidates (**Table 1**) were tested over 4 weeks based on the historical steroid and anti-TNFα clinical data and 5 followed a new direction testing for 12 weeks. The one successful report of a BTK inhibitor showing efficacy in RA patients was with fenebrutinib, the most selective BTK inhibitor in this class (**Table 4**) and was tested over this longer 12 week period (74). In addition, there was an improvement in efficacy with time of treatment observed with this BTK inhibitor. Fenebrutinib showed significant, but modest impact at 4 weeks that further increased at 8 weeks and reached its best impact at 12 weeks. Interestingly, this is a highly specific noncovalent BTK inhibitor and demonstrates that selectivity and good drug characteristics can compete with covalent inhibitors. A closer look at the 4 week RA trial done with spebrutinib as a typical short course BTK inhibitor trial in RA supports this length of trial confounding variable (67). Although spebrutinib did not demonstrate efficacy in RA, it displayed a strong trend towards improvement in RA clinical efficacy measures as well as a reduction in circulating bone resorption biomarkers, near complete BTK coverage, and a significant reduction in circulating transitional B cells and an increase in naive cells. This trial was stopped early due to slow enrollment and finished with half the cohort number projected to be necessary for demonstrating clinical efficacy. It is likely that this trial would have been successful if powered sufficiently as originally planned or run for a longer period. It should be noted that of the 4 BTK inhibitors reporting results publicly, 2 negative RA trials were at 4 weeks and 2 were at 12 weeks. While the 2 negative 12 week trials (BMS-9846142, evobrutinib) failed to meet statistical significance, there were trends toward improvement as reflected by increasing percentages of subjects in the dose groups *versus* placebo achieving low disease scores measures (ACR20, ACR50, ACR70, DAS28). Moreover, in the case of BMS-986142, the percentages of subjects achieving ACR20 scores increased with time. Given the positive fenebrutinib results at 12 weeks, the lack of reported success for MS-986142 and evobrutinib at 12 weeks may reflect other variables such as a need for longer trials and responder patient population subsets being under represented. The latter underscores the need for improvements in precision medicine and expanded biomarker measures that may refine selection of patients treated with BTK inhibitors.

### BTK Inhibition and Precision Medicine in Rheumatoid Arthritis

If disruption of B cell differentiation and its impact on subsequent processes in RA are the mechanism by which BTK inhibitors demonstrate efficacy, this may ultimately prove to be a patient selection criteria in precision medicine for determining appropriate therapy in RA. It has been proposed that the RA population can be further subcategorized based on gene signatures of the synovium underlying the immune pathology, representing lymphoid, myeloid, low inflammatory, and fibroid defined gene sets (75). High levels of B cell infiltrates are observed in the synovium of the lymphoid phenotype, moderate levels in the myeloid phenotype and are absent in the low inflammatory and fibroid phenotypes. Moreover, the anti-TNFα biologic responsive group was predominantly myeloid and the anti-TNFα biologic resistant group was predominantly lymphoid. Interestingly, a similar result is seen in mice. A recent study investigating the role of BTK in a mouse RA model demonstrates that lack of BTK reduces B cell differentiation and prevents arthritis development whereas immune-complexes that activate Fc receptors and inflammatory processes on myeloid cells are still able to induce arthritis in these mice (76). In patients with RA, B cells are known to play a major role in the pathology since depletion of B cells with Rituximab leads to significant improvement in the pathology (1) and relapse of arthritis post treatment is closely associated with the regeneration of B cells and plasmablasts (77, 78). Furthermore, post depletion improvement in RA correlates with reduction of B cell derived plasma cells in the synovium (8). It is possible that BTK inhibitors may also have some impact on myeloid type RA since low levels of B cells are present in the synovium with the myeloid cells (75). BTK inhibitors may also have direct effects on myeloid cells since BTK is known to regulate myeloid lineage differentiation as well as myeloid Fc receptor/antibody mediated inflammatory functions (12). Similar to B cells, if myeloid cells in RA are directly impacted by BTK, it may take time to observe disease impact, with potentially some level of downstream effects observed on inflammatory signaling processes. Taken together, these observations suggest that B cells and potentially myeloid lineage cells drive the pathology of RA more by cellular development and differentiation mechanisms than by inflammation. In addition, patients with lymphoid and potentially myeloid RA could represent a precision medicine approach to match BTK inhibitor therapeutic mechanism of action to disease specific pathology.

### BTK Inhibitors in Autoantibody Associated Diseases

As an extension to the theme of targeting B cell autoimmune indications with BTK inhibitors, four disease indications thought to be driven by autoantibodies (MS, PV, ITP, and SCU) have been or currently are being targeted in clinical trials. The treatment trial times in these studies occur over an extended period ranging from 16 to 108 weeks. The extended period of drug exposure is to allow enough time for autoantibody levels to drop below pathologic levels, both by clearance of circulating autoantibodies and by halting the continuing cycle of activated B cell conversion to new autoantibody producing plasmablasts and plasma cells. Results have been promising. MS, PV, and ITP have all been shown to respond to BTK inhibition in phase 2 clinical trials and have progressed to phase 3 testing (**Table 1**). Increasing evidence implicates B cells in the pathological development of MS (79). Consistent with this B cell mechanism hypothesis, the BTK inhibitor evobrutinib was shown to reduce autoantigen activation by self antigen binding to BCRs, maturation, cytokine production, and antigen presentation by B cells and thereby reduce the severity of experimental autoimmune encephalomelits (EAE) pathology in mice (80). Tolebrutinib was also shown to reduce EAE in a MOG peptide model of EAE (61). In MS clinical trials, both evobrutinib (81) and tolebrutinib (82) have demonstrated clinical efficacy in phase 2 trial of relapsing-remitting MS. Another autoantibody mediated disease, PV, is a condition with severe painful blistering on the skin and mucous membranes caused by autoantibodies against the cell-cell adhesion proteins desmoglein 1 and 3 (83). Consistent with the hypothesis that autoantibody driven diseases will benefit from BTK inhibition, Tolebrutinib has shown significant effects on reducing skin lesions in this condition (84). ITP is also thought to be driven by an autoantibody driven process resulting from the binding of autoantibodies to platelet surface antigens that cause aggregation, inflammation, and reduced platelet counts (85). In a study of ITP patients who were refractory to multiple anti-inflammatory treatments with no alternative therapeutic options, the BTK inhibitor rilzabrutinib demonstrated improvement as measured by an increase in platelet counts in subjects treated for a median period of 10 weeks (86). CSU is also thought to be caused by antibody driven mechanisms that results in pathogenic activation of mast cells and basophils by IgG and IgE autoantibodies (87). The noncovalent BTK inhibitor fenebrutinib, however, reported lack of efficacy in a CSU trial, while the covalent BTK inhibitor remibrutinib trial is ongoing. Covalent modification of BTK by remibrutinib treatment in healthy individuals is known to result in extended near complete covalent inhibition of BCR activation in skin basal cells (88). The results of the remibrutinib trial should help to determine whether autoantibody triggered basal cell activity is a key driver in this disease and whether extended BTK inhibition determines efficacy in CSU. While autoimmune diseases with autoantibody driven pathology mechanisms appear to be amenable to therapeutic intervention by BTK inhibitors, correlation of autoantibody threshold levels to clinical efficacy has not been published or well established in these studies. Future studies incorporating autoantibody measures are needed to clarify the role of BTK, BTK inhibition, and autoantibody levels with clinical efficacy.

### SLE and SJ Clinical Failures of BTK Inhibitors

Not all the autoimmune indications chosen for clinical testing of BTK inhibitors showed therapeutic benefit. SLE and SJ are two autoimmune diseases where BTK inhibition is not providing therapeutic benefit despite the presence of B cells in the associated pathology (89, 90). In the case of SLE, the lack of clinical impact observed by evobrutinib and fenebrutinib was unexpected given that nearly all BTK inhibitors in clinical trials showed efficacy in SLE mouse models for the disease. Changes in the observed biological biomarkers measured in SLE trials were consistent with the predicted changes derived from the SLE animal model studies. Fenebrutinib treatment of SLE for 48 weeks demonstrated the expected reduction in B cells, plasmablasts, IgG levels, and autoantibodies, but these did not translate into meaningful improvement of clinical measures (28). Similarly, Evobrutinib failed to show clinical efficacy over a 52 week treatment period (29). BTK inhibitors in the clinic for SJ have not reported data from animal models (if they were performed), but the decision to test in SJ is supported by knowledge of BTK’s role in autoimmune processes, that BTK is overexpressed in B cells of SJ patients (91), and that autoimmune antibodies are a hallmark of SJ (92). Treatment of SJ with the BTK inhibitors branebrutinib and tirabrutinib, however, did not show clinical benefit (**Table 1**). Lack of clinical benefit of BTK may have been expected since B cell clinical pathology contribution was called into question when a study with rituximab depletion of B cells was reported to not have therapeutic benefit in SJ (93). Though some biological pathology may be driven by BTK and B cells in SLE and SJ, as is evidenced by BTK inhibition altering pathology associated with B cells, its involvement in clinically meaningful pathology appears to be limited, reducing any potential therapeutic benefit of BTK inhibitors.

### Highlighting the Role of BTK in B Cell Trafficking and Differentiation

BTK is not reported to have genetic evidence linking it with the autoimmune disorders being clinically tested with BTK inhibitors, but functional signaling pathways associated with clinical pathology have individual proteins that themselves interact with signaling pathways connecting to BTK (**Table 3**). One example of this relationship is between CXCL12 as a driver of autoimmune diseases and its role in activating BTK as part of the chemotaxis process. The yellow highlighted interactions on the “chemotaxis_CXCL12/CXCR4-induced chemotaxis” pathway (**Figure 1**) illustrate the activated signaling cascade initiated by CXCL12 through the CXCR4 receptor and onto BTK (94). This activation of BTK leads to phosphorylation and activation of PLC-γ2 (15, 95). The pathway map illustrates the signaling cascade from PLC-γ2 to the activation of alpha-L/beta-2 integrin, which promotes the cell adhesion for leukocyte chemotaxis. BTK activation of PLC-γ2 similarly leads to the activation of alpha-4/beta-1 integrin, which also leads to cell adhesion and leukocyte chemotaxis (15, 96).

Continuing the hypothesis that CXCL12 is a driver of autoimmune diseases, a multi targeted approach for inhibiting BTK and other kinases could have beneficial effects. ITK and JAK3, two common off targets of BTK inhibitors, are also present on this pathway map. The involvement of ITK and JAK3 is illustrated as a series of molecular interactions that lead to cytoskeletal remodeling and leukocyte chemotaxis due to CXCL12/CXCR4 signaling. In addition to regulating cytoskeletal elements involved in chemotaxis, BTK also regulates BCR-induced cytoskeletal remodeling involved in antigen processing and presentation, thereby serving as a common nodal point of regulation for these two functions in B cells (97, 98).This leaves the third branch of CXCL12/CXCR4 induced chemotaxis open through the translation of proteins involved with cell migration. Combinations with other kinase inhibitors, such as MEK and MAPK, could prove synergistic by inhibiting translation of proteins promoting chemotaxis (99).

The indirect connection of BTK to the disease-associated CXCL12 pathway can be seen by its connection to PLC-γ 2 where a gain-of-function mutation in PLC-γ 2 leads to SLE-like autoimmunity by altering B cell Ca2+ signaling (100). Another association of BTK to disease (arthritis) *via* the CXCL12 signaling pathway can be seen by BTK’s involvement in mediating osteoclast differentiation and bone resorption activity in a CIA mouse model that can be blocked by an antagonist to BTK (101). Similarly, the clinically tested drug candidate spebrutinib was also shown to prevent osteoclast differentiation of human osteoclasts from cultured myeloid progenitor cells and to reduce the levels of circulating bone resorption biomarkers due to osteoclast activity in RA patients (67). The effects of BTK inhibitors at reducing the osteoclast differentiation in RA is consistent with the hypothesis discussed previously in this paper that BTK inhibitor efficacy requires longer treatment periods in order to reflect processes of B-cell and myeloid cell differentiation rather than the shorter times used to measure steroids and anti-TNFα biologics treating inflammation. The CXCL12 signal pathway is further support for the utility of BTK inhibitors in treating autoimmune diseases listed in **Table 1** since CXCL12 is associated with SLE, SJ (102–104), RA (105–108) and MS (109, 110). Even though CXCL12 was part of SJ and MS biomarker list, but not part of SLE and RA biomarker list, it could be a mediator as both CXCR4 and CD45 have genetic associations with SLE and RA.

Clinical indication selection for BTK inhibitor applications is often supported by pathway analysis to connect known signaling pathways associated with disease pathology to pathways where BTK has connections to this pathway but may not be genetic evidence linking it with the disease. An example for BTK connections to pathways associated with a normal cellular function also associated with disease, is the IgE receptor and CSU pathology. The IgE receptors expressed on the surface of basophils and mast cells are activated by the binding of IgE-antigen complexes that lead to phosphorylation and activation of SYK, which in turn phosphorylates and activates BTK (111–113). This cascade leads to the degranulation of mast cells and basophils. The connection of BTK to the degranulation of basophils was demonstrated in a study showing that BTK inhibition blocks degranulation (114). The connection of BTK to CSU is the IgE receptor overactivity implicated in the observed skin pathology (87). This pathway connection is one of the reasons that CSU is being therapeutically evaluated by remibrutinib and fenebrutinib in the clinic with results pending completion (**Table 1**).

### BTK Inhibitors Improving Selectivity and Changing Modes of Action

The selectivity of BTK for the seven targets we looked at has some interesting trends. JAK3 and EGFR inhibition has been eliminated in most second generation BTK inhibitors, except for a few examples like tolebrutinib, spebrutinib, and poseltinib. In the TEC kinase family, most BTK inhibitors studied here have improved their selectivity to be less potent against ITK when compared against ibrutinib. Selectivity against TEC and TXK is mixed, where over half of the second generation have reported low nanomolar IC_50_s against ibrutinib’s low-mid nanomolar inhibition. As the strength of BTK inhibition decreases, these inhibitors appear to become more selective in their target inhibition. The one exception to this trend is fenebrutinib, a noncovalent BTK inhibitor.

Selectivity against BMX would appear the most challenging off-target for these BTK inhibitors. The majority of these BTK inhibitors share a similar low nanomolar inhibition of BMX, as does ibrutinib. Only four (BMS-986142, tirabrutinib, acalabrutinib, evobrutonib) had IC_50_s in the low-mid nanomolar range. Fenebrutinib was the only inhibitor with a IC_50_ in the high-mid nanomolar range for BMX. But, selectively excluding BMX may not be as necessary since studies have investigated its involvement with inflammatory, cardiovascular diseases, and oncology (115–117). The off target inhibition of BMX by BTK inhibitors could have therapeutic efficacy as well. For instance, BMX has increased expression in cardiac endothelium as a response to ischemia and is being looked at as a possible treatment for cardiovascular diseases (118–121). It was also found that BMX regulates the TLR4 induced expression and TNF and IL-6, but IL-6 expression was independent of BTK inhibition of the p38 MAPK pathways (122). This raises the possibility that inhibition of BTK and BMX together may have synergistic effects as an anti-inflammatory treatment.

For the noncovalent BTK inhibitors, an interesting story is evolving around their selectivity. In general, both BMS-986142 and fenebrutinib are more selective than ibrutinib as both have IC_50_s for JAK3 and EGFR in the high nanomolar range. The distinction between these two reversible inhibitors is in their activity against the TEC family kinases. Fenebrutinib is the most selective having high nanomolar IC_50_s for TEC, ITK, and TXK and high-mid nanomolar for BMX. BMS-986412 has reported a high-mid nanomolar IC_50_ for ITK and low-mid nanomolar inhibition of TEC, BMX, and TXK. Both reported BTK inhibition in the low nanomolar range.

One interesting comparator from this analysis will be the differences and similarities between a selective covalent BTK inhibitor like elsubrutinib and a selective noncovalent inhibitor like fenebrutinib when it comes to patients that have mutations at cysteine 481. Both the covalent BTK inhibitor elsubrutinib and the noncovalent inhibitor fenebrutinib would appear, disregarding the lack of data for BMX inhibition for elsubrutinib, to be selective for BTK, with some potency for BMX inhibition. The covalent inhibitor elsubrutinib appears to have paid for this selectivity with reduced BTK potency. In contrast, fenebrutinib may have achieved its selectivity by no longer being locked into chemical structure requirements for forming the covalent bond with cysteine 481.

Enhancement of binding specificity with noncovalent BTK inhibitors when achievable may have advantages when it comes to addressing human population subsets with BTK mutations. It has been found that cancer patients treated with BTK inhibitors may acquire resistance by BTK mutations at cysteine 481, which is required for covalent binding (123–126) and poses a problem for covalent BTK inhibitors in these subpopulations. As discussed in the section on “Clinical Study Paradigms and Covalent BTK Inhibition”, covalent BTK inhibitors were optimized for cysteine 481 bond formation and the resulting long term enzyme inhibition to effectively impact B cell maturation and myeloid and B cell activation processes. While this covalent BTK inhibition mechanism provides an advantage over noncovalent inhibitors in terms of effective inhibition of BTK long after circulating drug has cleared the body, most if not all covalent BTK inhibitors were not optimized for typical good drug characteristics such as sufficient circulating exposure times in the blood, potent BTK noncovalent binding affinity, and slow enzyme dissociation rates. As a result, cysteine 481 mutant BTK would be expected to not bind to or be exposed to covalent BTK inhibitors sufficiently to have therapeutic effects at a drug dose effective with normal cysteine 481 containing BTK. This may negatively impact drug efficacy in autoimmune indications where extensive long term exposure appears to be required based on current clinical trials such as observed in RA. In principal, this concept has been shown in B cells derived from a mouse model containing a serine mutation for cysteine 481 in BTK that demonstrated impaired BTK signaling and B cell activation after BCR stimulation when cells were pretreated with the covalent BTK inhibitor ibrutinib, whereas inhibition was still achieved by pretreatment with the good binding characteristics of the noncovalent inhibitor RN486 (127). To date, no data has been reported for BTK inhibitors currently in autoimmune disease clinical trials regarding the effects of BTK cysteine 481 mutations on drug efficacy or whether such mutations are common in the autoimmune diseases studied with these drugs. Given the successful trials of the noncovalent BTK inhibitor fenebrutinib in RA and the covalent BTK inhibitors tolebrutinib in MS and rilzabrutinib in ITP, it appears that that both types of BTK inhibitors have a future in autoimmune therapeutic treatment. Moreover, in those cases where resistance to covalent BTK inhibitors due to cysteine 481 mutations do arise, there will be noncovalent BTK inhibitor treatment options available.

### Continuing Improvement in BTK Inhibitor Safety Profiles

Improvements in selectivity of BTK inhibitors have improved safety profiles since the development of ibrutinib, but there is still much work to be done. Atrial fibrillation is one of the most reported adverse events for ibrutinib and generally one of the top three concerns pushing for more selectivity in BTK inhibitors. Even with the improved selectivity of acalabrutinib, regulatory agencies have confirmed this adverse event persists. While drug off target causation of arrhythmias, such as atrial fibrillation, is complex, one possible factor mediating this activity is with interference of class I phophoinositide 3-kinase isoforms involved in cardiac hypertrophy, contractility, and regulation of various channel forming proteins (128). In a retrospective cohort study with Chronic lymphocytic leukemia (CLL) atrial fibrillation was the most common cause of ibrutinib discontinuation (129). Clinical studies comparing acalabrutinib and ibrutinib in CLL patients have found relatively similar safety profiles for both therapies, with only a minor note of increased severity of headaches for acalabrutinib (130). More recent interim analysis of a randomized phase 3 ALPINE study comparing the oncology approved drugs ibrutinib and zanubrutinib in CLL/SLL patients found zanubrutinib had lower rates of atrial fibrillation than ibrutinib (131). Comparing the off-target inhibition of phosphoinositide 3-kinase by second generation BTK inhibitors could provide more insight into possible improvements to this safety event, but no publications have come out with IC_50_s for this off target other than for ibrutinib. Future research into this relationship could unveil new avenues for optimizing the cardiac safety profile by improving selectivity for PI3K.

Another major adverse side effect of ibrutinib is bleeding (132), including major hemorrhages. This bleeding is thought to be due to a combination of on-target irreversible BTK inhibition, as well as off-target inhibition of other kinases, including EGFR, ITK, JAK3 and TEC kinase. The effect of Ibrutinib on bleeding could be multifactorial but the level of TEC inhibition may govern the bleeding risk because TEC kinase phosphorylation is highly dependent on platelet aggregation (133). While ibrutinib is known to irreversibly inhibit both BTK and TEC, acalabrutinib shows a higher specificity towards BTK and less activity on TEC (**Table 4**) (65, 133, 134). Ibrutinib has also been shown to inhibit Src-kinases in washed human platelets, they play an important role for platelet activation and are upstream of BTK and TEC in the glycoprotein VI signaling pathway (135). Src-kinase inhibition has been shown to induce bleeding *in vivo* (136). Studies show acalabrutinib seems to have less inhibitory potential than ibrutinib on Src-kinase (134, 137). Future results from clinical studies on BTK inhibitors with weak TEC inhibition such as fenebrutinib and elsubrutinib will shed more light on how effective their improved selectivity will benefit the occurrence of bleeding in the safety profile.

While still in the middle of their clinical development for treating autoimmune diseases, results from some of the most selective BTK inhibitors look promising as mild to moderate safety events are most commonly reported. Ph II study of evobrutinib in 469 patients with Systemic lupus Erythematosus did not report bleeding and atrial fibrillation as adverse events, which are concerns with ibrutinib. Most frequently reported adverse events that were non-serious included urinary tract infection, diarrhea, nasopharyngitis and upper respiratory tract infection (138). For fenebrutinib, a Ph II study comparing fenebrutinib and adalimumab, a TNF-alpha inhibitor, in patients with rheumatoid arthritis had commonly reported non-serious adverse events of nausea, headache, anemia and upper respiratory tract infections (74). While serious adverse events including pneumonia, pyelonephritis and cellulitis were reported, they are still under review by regulatory authorities. A limitation of this analysis is the need for publicly reported safety data from pre-clinical testing and clinical studies. Pre-clinical safety data is rarely published publicly and as many of these drugs are currently going through clinical trials and are not launched yet, they lack reported outcomes from large patient cohorts. Another limitation is that covalent BTK inhibitors continue to face challenges in improving selectivity within the TEC family of kinases. While further data is needed to confirm all types and severity of the adverse events for these selective BTK inhibitors, they do show promise in reducing the occurrence of the most severe adverse events related to ibrutinib.

### Oncology BTK Inhibitor Indication Expansion to Autoimmune Diseases

The BTK inhibitors discussed in this paper that are being tested in the clinic to treat autoimmune diseases and if approved, will be the first in class drugs for treating these diseases. Follow on BTK inhibitors will seek to displace them by demonstrating better efficacy, safety, and tolerability. BTK inhibitors launched or being developed in cancer have extensive clinical safety and efficacy data already established and that will allow for efficient expansion into autoimmune disease indications, bypassing typical Phase 1a/b activities and moving directly into phase 2 or event phase 2/3 trials. For example, the covalent BTK inhibitor zanubrutinib, which is approved by the FDA for use in Waldenström’s macroglobulinemia, mantle cell lymphoma, and marginal zone lymphoma, announced the initiation of clinical trials to test its efficacy in active proliferative lupus nephritis late in 2020, anticipating study completion in August of 2023 (clinicaltrials.gov identifier NCT04643470). The covalent BTK inhibitor orelabrutinib was approved in China for relapsed/refractory chronic lymphocytic leukemia, small lymphocytic lymphoma, and relapsed/refractory mantle cell lymphoma and, after having received breakthrough designation for cancer treatment with the FDA this year, could be another candidate for indication expansion into the autoimmune space. Noncovalent BTK inhibitors such as pirtobrutinib (LOXO-305), MK-1026 (ARQ-531), fenebrutinib (GDC-0853), and vecabrutinib (SNS-062) also look promising in oncology clinical trials (139) and may similarly be candidates for treating autoimmune diseases.

## Conclusions

Collectively, the new class of BTK inhibitors show promise in bringing new treatment paradigms to treating autoimmune disease disorders. Enhancement of selectivity, unique enzyme coverage characteristics, and pharmacokinetic profiles has improved safety margins for most members in this BTK group of clinical drug candidates. Recent success in clinical trials treating antibody mediated diseases such as MS, PV and potentially RA appear to be most promising, while broader less causally understood disease like SLE and SJ appear not to be of therapeutic benefit. Taken together, major improvements in target selectivity are leading to the reduced observance of serious adverse events while maintaining clinical efficacy shows great promise for the feasibility for the next generation BTK inhibitors in providing a safe and efficacious treatment for autoimmune patients.

## Author Contributions

GR contributed to the research and writing of this manuscript. MW contributed to the research and writing of this manuscript. KO contributed to the research and writing of this manuscript. All authors contributed to the article and approved the submitted version.

## Conflict of Interest

Author GER was employed by company Eisai Inc. MW and KO are employed by Clarivate that owns the commercial database products used in the research presented.

## Publisher’s Note

All claims expressed in this article are solely those of the authors and do not necessarily represent those of their affiliated organizations, or those of the publisher, the editors and the reviewers. Any product that may be evaluated in this article, or claim that may be made by its manufacturer, is not guaranteed or endorsed by the publisher.

## Supplementary Material

Supplementary Table 1 Full enrichment excel file.

Supplementary Table 2 Full OFF-X excel file with drug score and # of alerts.
